# The Relation of Dental Caries to Certain Forms of Indigestion

**Published:** 1894-02

**Authors:** E. F. Wilson

**Affiliations:** Columbus


### The Relation of Dental Caries to Certain Forms of Indigestion.

BY E. F. WILSON, M.D., COLUMBUS.

Read before the Ohio State Dental Society, December, 1893.

To the general practitioner of medicine no subject is of more importance than that of the various disorders classed under the head of Indigestion or Dyspepsia. That they have always been the "bete noire" of the profession is shown by the infinite number of remedies that have been, from time to time, heralded as cures. The patients of this class are the ones who wander from one physician to another with their tale of woe, and tell each one in turn how they have been treated or mistreated by his predecessor.

Volumes have been written on the subject, and it has been the aim of hundreds of investigators to find what the causes are that underlie these troubles. There are, no doubt, a variety of causes, and in this, as in every thing else, each cause produces its own effect.

I wish to-day to call your attention, for a short time, to one cause that seems to me of great importance in producing certain classes of these disorders. It is also a cause that is very often overlooked or neglected. That the condition of the teeth should have a great deal to do with the subject of indigestion is apparent to all. Every physician has seen the old man or old woman, without any teeth, or only a few "snags," complaining all the time of trouble with the digestive apparatus. They say their stomachs hurt them and that they have to "rift" wind for several hours after eating. They are uncomfortable and unhappy. They would answer "no," if asked the question, "Is life worth living?" But see that same old man or woman after one of our friends, the dentists, had supplied him or her with a set of new teeth. They are entirely different persons, cheerful and happy so far as a fairly good digestion can make one.

But this trouble with the teeth is not confined to the old and toothless. It begins with the infant. Nature recognizes the fact that the stomach is not able, by itself, to digest any but the most easily assimilated food, so she furnishes the young child milk, and a milk that does not form a hard curd, but is more of a flocculent mass and very easily taken up and digested. As the teeth begin to come the salivary glands also begin to develop and prepare their secretion to assist in the digestion of mixed foods that the child is now ready for. In a large per cent. of children the teeth are not perfect and decay begins soon after they are erupted. Whenever this happens, almost without exception, the child will develop some one of the various symptoms that accompany digestive troubles in children. Until very recently the general practitioner of medicine has not attached much importance to the decay and loss of the deciduous teeth, and almost nothing has been done to encourage their preservation until such time as the permanent set is erupted.

The teeth stand at the entrance or gate of the body, and their chief duties are the mechanical part of digestion. If the teeth are imperfect or decayed the mastication of the food is imperfect. Instead of going to the stomach in fit condition to be acted on by the digestive ferments, it is more nearly in the condition of the food of birds, that have no teeth, but are supplied with a gizzard. Unfortunately in our make-up the gizzard was omitted, and the result is that if the teeth are not in condition to do this work properly and well, we suffer the pangs of indigestion, with all the sympathetic disorders that follow in its train.

It is true that the evil effects of "bad teeth" are seen more frequently in childhood and in old age than at any other time of life. I am satisfied that in children a number of diseases may be traced to the carious condition of the teeth. Suppuration of the middle ear is caused again and again by a carious tooth. Disease of the eye is also caused by the condition of the teeth. Various neuralgias and reflex nervous symptoms arise from the same cause. Last, but by no means least of all, come the digestive troubles brought on by the carious teeth.

Have these things been regarded sufficiently in the past? How many of you, until within the past few years, have been called upon to treat and preserve the "milk teeth?" Was it not, rather, is it not the fashion to let them go, as they will not pay for being treated, they will be replaced so soon by the permanent teeth? This false economy has been responsible for a countless number of sickly, ill-nurtured children, who if they live to reach puberty make the nervous, dyspeptic men and women that are met in every walk of life, and that are pointed out by our English cousins when they say "Americans are a race of dyspeptics."

The art and science of dentistry has reached such a high plane that any neglect of the children's teeth in the future will be little less than criminal. It is also to be hoped that the time has come when the physician will realize his obligation in this matter and will have the children's teeth carefully examined and treated at least once in six months. In regard to older patients it is hardly necessary to say anything. It is recognized, both by the patient and physician as necessary for æsthetic as well as for hygienic reasons, that the teeth should be kept in repair. There are though a large per cent. of people, who on account of their finances, or for some other reason, neglect the teeth and shun the dentist. It is among this class that we find the digestive trouble caused by faulty mastication and fermentation.

The troubles caused by faulty mastication are entirely mechanical and have already been referred to. Those of fermentation are still to be explained. Let us call your attention for a moment to the pathology of caries. Prof. Miller, of Berlin, says: "It has long been known that the human mouth was the abode of numerous microscopic organisms; but only within the last five or ten years, since more exact methods of bacteriological investigation have come into use, we have been able to acquire much definite knowledge as to the morphology, physiology, etc., of these minute organisms. Recently the study of the bacteria of the human mouth has been zealously pursued by various investigators, and much has been accomplished toward bringing to light the causes of various affections of the mouth and associate parts.

"There is no part of the human organism which furnishes a more universal culture medium for bacteria than the oral cavity, and the different varieties of micro-organisms that find in it conditions favorable to their development are correspondingly numerous. Of these the larger part are of a non-pathogenic character, existing upon the various organic substances in the oral secretions, and upon particles of food which have been allowed to remain between the teeth, but producing no particular deleterious effects upon the surrounding parts other than the decay of the teeth." Miller divides the oral bacteria into several classes, one of which is composed of a large number of bacteria that decomposes sugar, forming lactic acid. It has been proved in the past few years, that decay is caused by a decalcification of the tooth substance, followed by a solution of the decalcified basis substance. The decalcification is brought about by acids, chiefly lactic acid; and these acids are produced by the action of micro-organisms on carbohydrates. The solution of the decalcified tooth substance is accomplished by the peptonizing power of the same or other micro-organisms. So much for the pathology of caries.

What bearing has all this on our subject? Can these micro-organisms be carried to other parts of the body and there do harm? James Israel describes a number of cases of abscess on the neck, chronic pyæmia, etc., which owed their origin to these organisms. That they are also able to produce both chronic and acute disturbances of digestion there can be no doubt. By the continual swallowing of the bacteria lodged in the mouth we have one of the most frequent causes of fermentation dyspepsia. It is now known that most of the bacteria found in the mouth may pass through the stomach without losing their vitality, and under certain circumstances may produce severe disturbances in the intestinal tract. So, now that the pathology of dental caries is better understood, the connection between the proper care of the teeth and some of the phenomena of indigestion are easily traced.

To us, as practitioners of medicine and dentistry and as practical men, all these things show only this: what an important field we have in the human mouth for investigation and study; and how much the general health depends on the proper care of the teeth.

